# Impact of a deep learning assistant on the histopathologic classification of liver cancer

**DOI:** 10.1038/s41746-020-0232-8

**Published:** 2020-02-26

**Authors:** Amirhossein Kiani, Bora Uyumazturk, Pranav Rajpurkar, Alex Wang, Rebecca Gao, Erik Jones, Yifan Yu, Curtis P. Langlotz, Robyn L. Ball, Thomas J. Montine, Brock A. Martin, Gerald J. Berry, Michael G. Ozawa, Florette K. Hazard, Ryanne A. Brown, Simon B. Chen, Mona Wood, Libby S. Allard, Lourdes Ylagan, Andrew Y. Ng, Jeanne Shen

**Affiliations:** 1 Department of Computer Science, Stanford University, Stanford, CA USA; 2 Stanford University School of Medicine, Stanford, CA USA; 3 Center for Artificial Intelligence in Medicine & Imaging, Stanford University, Stanford, CA USA; 4 Department of Radiology, Stanford University, Stanford, CA USA; 5 Department of Pathology, Stanford University, Stanford, CA USA

**Keywords:** Pathology, Machine learning, Liver cancer

## Abstract

Artificial intelligence (AI) algorithms continue to rival human performance on a variety of clinical tasks, while their actual impact on human diagnosticians, when incorporated into clinical workflows, remains relatively unexplored. In this study, we developed a deep learning-based assistant to help pathologists differentiate between two subtypes of primary liver cancer, hepatocellular carcinoma and cholangiocarcinoma, on hematoxylin and eosin-stained whole-slide images (WSI), and evaluated its effect on the diagnostic performance of 11 pathologists with varying levels of expertise. Our model achieved accuracies of 0.885 on a validation set of 26 WSI, and 0.842 on an independent test set of 80 WSI. Although use of the assistant did not change the mean accuracy of the 11 pathologists (*p* = 0.184, OR = 1.281), it significantly improved the accuracy (*p* = 0.045, OR = 1.499) of a subset of nine pathologists who fell within well-defined experience levels (GI subspecialists, non-GI subspecialists, and trainees). In the assisted state, model accuracy significantly impacted the diagnostic decisions of all 11 pathologists. As expected, when the model’s prediction was correct, assistance significantly improved accuracy (*p* = 0.000, OR = 4.289), whereas when the model’s prediction was incorrect, assistance significantly decreased accuracy (*p* = 0.000, OR = 0.253), with both effects holding across all pathologist experience levels and case difficulty levels. Our results highlight the challenges of translating AI models into the clinical setting, and emphasize the importance of taking into account potential unintended negative consequences of model assistance when designing and testing medical AI-assistance tools.

## Introduction

The rapid rate of scientific discovery in pathology has prompted a trend toward subspecialization,^1–3^ which has made it more difficult for general surgical pathologists to maintain expertise across the entire range of diverse specimen subtypes.^4–6^ This trend has resulted in misalignment of expertise for subspecialists, when reviewing specimens outside of their area of focus.^7,8^ Such situations are commonly encountered, for example, during after-hours intraoperative consultations, when pathologists are confronted with a diverse set of cases across a range of specimen subtypes. A lack of access to subspecialty expertise in this context can slow diagnostic turnaround times, resulting in delays in patient care and potential adverse impacts on clinical outcomes.

In recent years, advances in the field of artificial intelligence (AI) have led to the development of high-performance algorithms for a wide range of diagnostic tasks in medicine.^9–12^ One commonly encountered task is histopathologic tumor classification, which is critical for prognosis and treatment, and may be challenging, even for subspecialty-trained pathologists.^13–16^ To date, AI algorithms have achieved high accuracy on some tumor classification tasks.^17,18^ However, much of the work in this area has focused on the retrospective evaluation of model performance on ground-truth-labeled validation datasets. Few studies have taken the next step of evaluating the impact of model assistance on pathologist diagnostic performance.^19^ Furthermore, most recent applications of AI assistance to pathology have focused on models which run on whole-slide images (WSI) in a completely automated fashion, independent of human input, prior to the post-analytical stage. This creates a barrier to access for the majority of pathology practices, particularly in global health settings, which lack the expensive digital slide scanners necessary for generating the WSI on which these models run. Even when slide scanning services are readily available, the additional turnaround time necessary to digitize cases for input into an AI model makes these tools impractical for use in clinical diagnostic workflows, unless the practice has already adopted a completely digital workflow. For time-sensitive tasks, such as tumor classification, a preliminary diagnostic impression must be reached quickly on examination of routine hematoxylin and eosin (H&E)-stained slides, as many downstream ancillary tests (immunohistochemistry, fluorescence in situ hybridization, and molecular testing, for example) depend on this preliminary impression. Therefore, an AI diagnostic assistant for use in the average pathology lab should be easily accessible, quick, and able to accept as input any type of digital pathologic image, not just WSI. This might necessitate a degree of pre-analytical input by pathologist end users—for instance, in selecting the specific regions of interest on a slide that should be captured for analysis. While machine learning models for non-WSI digital analysis have been used for many years in research settings, they have seen less application in clinical settings, and little is known about the dependence of model performance and clinical utility on user background and diagnostic experience level.

In an effort to address these gaps, we built and tested a diagnostic support tool to help pathologists distinguish between hepatocellular carcinoma (HCC) and cholangiocarcinoma (CC), the two most common types of primary liver cancer. HCC and CC account for 70 and 15% of cancers arising in the liver, respectively, and the diagnostic distinction between these entities has unique implications for prognosis and patient management.^20^ For example, orthotopic liver transplantation is a widely accepted treatment for patients with HCC, but is often contraindicated in patients with CC. Distinguishing between the two may be challenging, even for subspecialized gastrointestinal (GI) pathologists.^21–23^

The diagnostic assistant presented in this work consists of a cloud-deployed AI model and a browser-based interface, where pathologists can receive a virtual second opinion in real time. The model was developed and trained using a publicly available dataset of H&E-stained digital WSI of HCC and CC. Using an independent test dataset, we evaluated the effect of the assistant on the diagnostic accuracy, sensitivity, and specificity of 11 pathologists with varying levels of relevant expertise, and explored the impact of pathologist experience level, case difficulty level, and model accuracy on the pathologists’ diagnostic performance with the use of the assistant.

## Results

### Diagnostic assistant development and performance evaluation

**Fig. 1:**
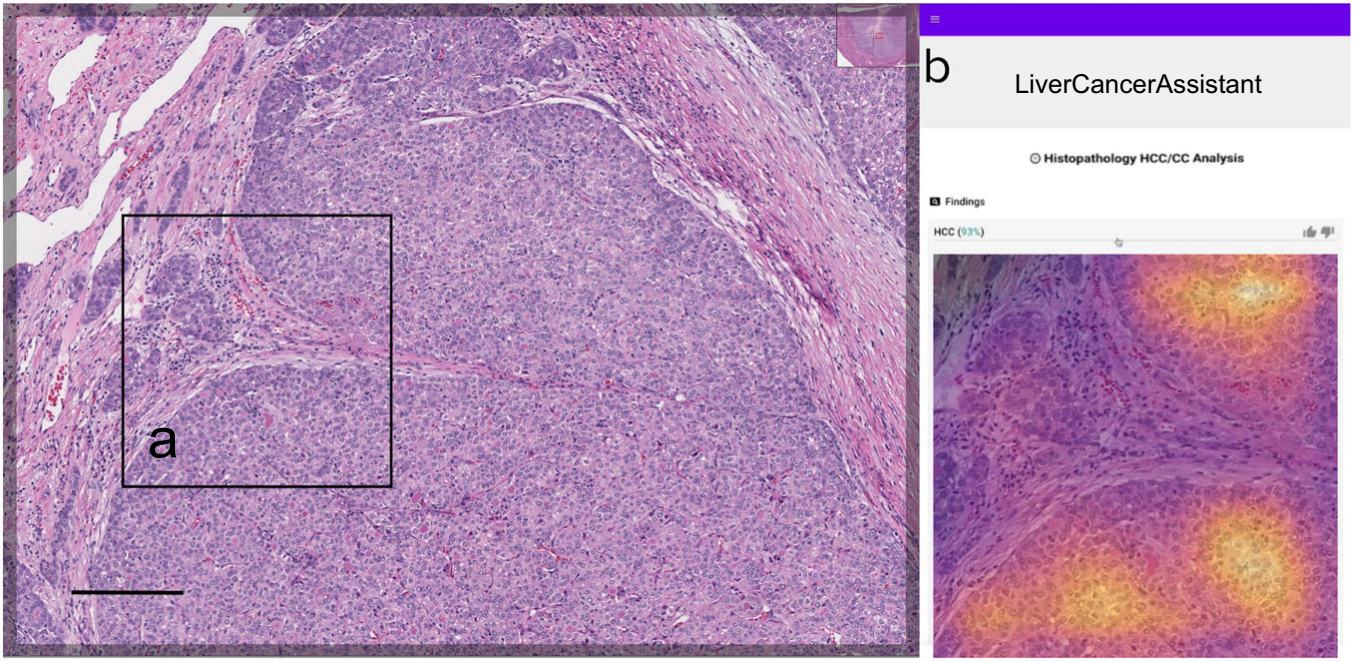
Graphical user interfaces for the experiment. The pathologists navigated the slide using the ObjectiveView image viewer. After identifying a tumor region of interest (ROI), they saved a 500 × 500 μm image patch at ×10 objective magnification (**a**) containing the ROI using the ‘crop’ tool. (The horizonal scale bar denotes 200 μm). After uploading the image patch to the diagnostic assistant’s user interface (**b**), they received a probability of each diagnosis (here, HCC), with an accompanying class activation map to assist with interpretation.

To develop the deep learning model for our web-based assistant, we used a total of 25,000 non-overlapping image patches of size 512 × 512 pixels (128 × 128 µm), extracted from tumor-containing regions from 70 (35 HCC and 35 CC) digital WSI of H&E-stained slides from formalin-fixed, paraffin-embedded (FFPE) primary hepatic tumor resections. These WSI were obtained from the Cancer Genome Atlas’ (TCGA) hepatocellular carcinoma (LIHC) and cholangiocarcinoma (CHOL) diagnostic FFPE WSI collections.^24^ The WSI were randomly partitioned into training (20,000 patches from 20 WSI), tuning (2400 patches from 24 WSI) and validation (2600 patches from 26 WSI) datasets, maintaining a 50:50 distribution of HCC and CC examples within each dataset. A convolutional neural network (CNN)^25^ with a DenseNet architecture, which has been shown to outperform traditional CNNs on image classification tasks,^26^ was incorporated into a simple prototype diagnostic support tool (see Methods) consisting of a web interface where pathologists could upload H&E image patches and receive the model’s predictions and explanatory class activation maps^27^ (CAMs) in real time (Fig. 1). The model’s predictions were displayed as probabilities for each diagnostic category (HCC and CC), with CAMs displayed as heatmaps highlighting the image regions most consistent with each respective diagnosis (Supplementary Fig. 4). To assess the model’s generalizability to previously unencountered data, we tested its performance on the internal validation dataset of 2600 (TCGA) image patches, by averaging the individual patch-level probabilities across all 100 patches randomly extracted from segmented tumor regions on that WSI, and converting this average to a binary slide-level prediction, using a probability threshold of 0.5. These slide-level predictions were then compared with the ground-truth labels to calculate the diagnostic accuracy of the model. Using this method, the model achieved a diagnostic accuracy of 0.885 (95% confidence interval (CI) 0.710–0.960).

### Performance of pathologists with and without assistance

**Fig. 2:**
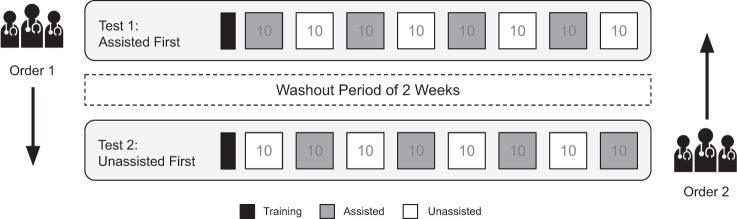
Experimental design. Each of the 11 pathologists was randomly assigned to either test Order 1 or 2. Each test began with a brief practice block of 4 (2 HCC and 2 CC) practice whole-slide images (WSI), followed by 8 experiment blocks of 10 WSI each, with Order 1 beginning with assistance and Order 2 beginning without assistance. The same 80 experiment WSI were reviewed in the same sequence during Tests 1 and 2, across both test Orders.

**Fig. 3:**
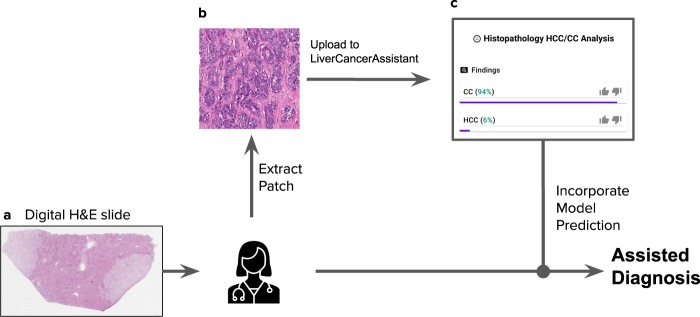
Pathologist diagnostic workflow with assistance. After reviewing the H&E whole-slide image (**a**), the pathologist extracts a tumor patch at ×10 objective magnification (**b**) and uploads it to the cloud-based model, which outputs predicted probabilities for cholangiocarcinoma (CC) and hepatocellular carcinoma (HCC) into the user interface (**c**), as well as corresponding class activation maps (not shown). These outputs are integrated with the pathologist’s diagnostic impression, to result in a final assisted diagnosis.

To assess the impact of our assistant on pathologist accuracy, we performed a study measuring the diagnostic accuracy of 11 pathologists, with and without assistance, who were classified into the following four experience level subgroups: GI subspecialty pathologists (*n* = 3), non-GI subspecialty pathologists (*n* = 3), pathology trainees (*n* = 3), and pathologists, not otherwise classified (*n* = 2) (for detailed subgroup definitions, see Methods). An independent test dataset of 80 WSI (40 CC and 40 HCC) of FFPE tumor tissue sections from Stanford University Medical Center was used for the study. Both the WSI used for model development and those used for the pathologist study were scanned at ×40 objective magnification (0.25 µm per pixel). Table 1 and Supplementary Fig. 1 summarize the datasets and the model development and selection process. To compare the performance metrics of the pathologists with and without assistance, each pathologist diagnosed the same test set of 80 WSI (presented in the same sequence) twice, in two separate sessions, according to the crossover design detailed in Fig. 2. During each session, they interpreted half of the study WSI with the assistance of the diagnostic support tool, and half without. The pathologists were blinded to the original diagnoses, clinical histories, and follow-up information. After a washout period of at least 2 weeks, per consensus recommendations from the College of American Pathologists for avoiding short-term memory bias in digital pathologic validation studies,^28^ the pathologists interpreted the same set of 80 WSI with the assistance status reversed; the WSI that were reviewed with assistance during the first reading were reviewed unassisted during the second reading, and vice versa. During the washout period, all pathologists were engaged in routine daily practice, which involved review of their usual clinical case mix. The pathologists were randomized into two groups, with one group beginning the diagnostic session with assistance, and the other without. The set of 80 WSI was divided randomly into 8 sets of 10 WSI. Within each set, the order of review of the WSI was identical. On unassisted cases, the pathologists reviewed each WSI in a digital whole-slide image viewer and recorded their diagnosis using a web-based survey interface. On assisted cases, they selected one or more patches from each WSI in the image viewer, saved as PNG files that they uploaded into the assistant for real-time analysis (Fig. 1). After viewing the assistant’s outputs, which were available within a few seconds, they recorded their final diagnosis using the web-based survey. The pathologist workflow is detailed in Fig. 3.

**Table 1:** Dataset and patient characteristics.

Source	Dataset	Class	No. of slides	Median patient age^a^	No. of female patients^b^
TCGA	Total	HCC	35	57.0 (17.0)	11 (31.4)
		CC	35	64.0 (13.0)	20 (57.1)
	Training	HCC	10	56.5 (13.5)	1 (10.0)
		CC	10	65.0 (10.75)	8 (80.0)
	Tuning	HCC	12	63.0 (16.0)	5 (41.6)
		CC	12	71.0 (8.75)	6 (50.0)
	Validation	HCC	13	55.0 (21.0)	5 (38.5)
		CC	13	59.0 (10.0)	6 (46.1)
Stanford	Indep. test	HCC	40	64.5 (8.25)	10 (25.0)
		CC	40	63.0 (14.75)	14 (35.0)

^a^The interquartile range (IQR) is provided in parentheses.

^b^The percentage of female patients is provided in parentheses.

**Fig. 4:**
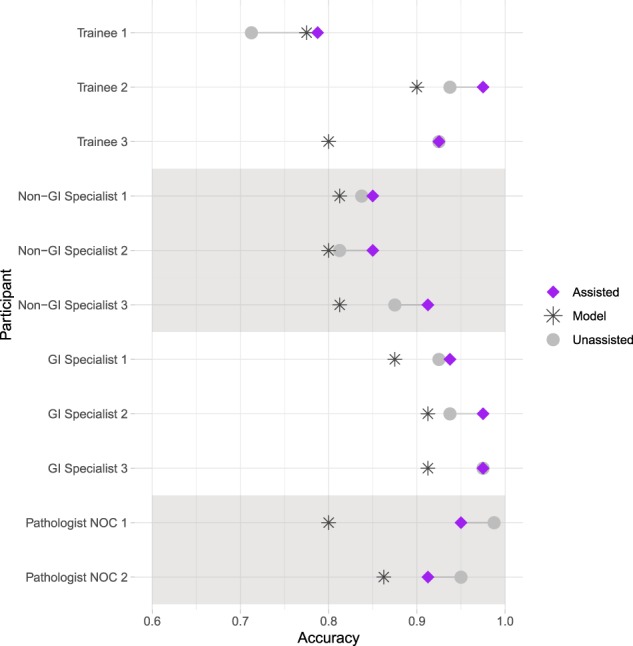
Impact of assistance on individual pathologist diagnostic performance. The average diagnostic accuracy (across the set of 80 experiment WSI) for each pathologist is plotted as follows: gray circle (unassisted) = accuracy of the unassisted pathologist, star (model) = accuracy of the model alone (based on pathologist selected input patches), purple diamond (assisted) = accuracy of the pathologist with model assistance.

The accuracy of the 11 pathologists, as a group, was 0.898 (95% CI 0.875–0.916) without assistance, and 0.914 (95% CI 0.893–0.930) with assistance. Among the pathologist subgroups, the accuracy of GI specialists was 0.946 (95% CI 0.909–0.968) without assistance, and 0.963 (95% CI 0.930–0.980) with assistance. The accuracy of non-GI specialists was 0.842 (95% CI 0.790–0.882) without assistance and 0.871 (95% CI 0.822–0.910) with assistance. The accuracy of trainees was 0.858 (95% CI 0.809–0.897) without assistance and 0.896 (95% CI 0.851–0.928) with assistance. The accuracy of pathologists not otherwise classified (NOC) was 0.969 (95% CI 0.929–0.987) without assistance and 0.931 (95% CI 0.881–0.930) with assistance. The number of correct diagnoses and mean accuracy for each pathologist subgroup, with and without assistance, is detailed in Table 2. The accuracies for individual pathologists, with and without assistance, are represented in Fig. 4 and Supplementary Table 1, and the corresponding sensitivities and specificities are detailed in Supplementary Figs 5 and 6.

**Table 2:** Pathologist unassisted and assisted accuracies, by experience level^a^.

Assistance	GI specialists	Non-GI specialists	Trainees	Pathologists NOC
Assisted	0.963 (0.930, 0.980) (*n* = 231)	0.871 (0.822, 0.910) (*n* = 209)	0.896 (0.851, 0.928) (*n* = 215)	0.931 (0.881, 0.961) (*n* = 149)
Unassisted	0.946 (0.909, 0.968) (*n* = 227)	0.842 (0.790, 0.882) (*n* = 202)	0.858 (0.809, 0.897) (*n* = 206)	0.969 (0.929, 0.987) (*n* = 155)

^a^The average accuracy of each pathologist subgroup, along with the 95% confidence interval (in parentheses) and number of correct diagnoses made (*n*) is presented.

When all 11 pathologists were included in a mixed-effect logistic regression model which controlled for the fixed effects of pathologist experience level and case difficulty level (using tumor grade as a proxy indicator), as well as the random effects of pathologist and WSI, there was no statistically significant increase in mean pathologist accuracy with assistance, for the group as a whole (*p* = 0.184, OR = 1.281, 95% CI 0.882–1.862). However, in a sensitivity analysis exploring the impact of assistance on the subset of nine pathologists of well-defined experience levels (GI specialists, non-GI specialists, and trainees) (Table 3 and Supplementary Table 4), we found that assistance resulted in a significant increase in mean accuracy (*p* = 0.045, OR = 1.499, 95% CI 1.007–2.230).

**Table 3:** Impact of assistance on diagnostic accuracy under different conditions^a^.

	Assistance (11 pathologists)	Assistance (9 pathologists)	Model correct (11 pathologists)	Model incorrect (11 pathologists)
OR (95% CI)	1.281 (0.882, 1.862)	1.499 (1.007, 2.230)	4.289 (2.360, 7.794)	0.253 (0.126, 0.507)
*p*-value	0.184	0.045	0.000	0.000

^a^The results of mixed-effect logistic regression analyses evaluating the impact of assistance on diagnostic accuracy are presented as odds ratios (OR) for pathologist diagnostic accuracy, with 95% confidence intervals (95% CI) and *p*-values from likelihood ratio testing (a two-tailed *p* ≤ 0.05 was considered statistically significant).

### Effect of pathologist experience level and tumor grade on diagnostic accuracy

The results of our mixed-effect model fit to the entire group of 11 pathologists indicated that, all other effects being the same, the pathologist experience level (*p* = 0.005) had a significant effect on diagnostic accuracy (Supplementary Table 3), with the odds ratios suggesting that non-GI specialists (OR = 0.204, 95% CI 0.082–0.508) and trainees (OR = 0.299, 95% CI 0.119–0.753) were less likely to make a correct diagnosis, compared with GI specialists. Similarly, when all other effects were held the same, the tumor grade (*p* = 0.010) had a significant effect on diagnostic accuracy, with the odds of making a correct diagnosis on grade 3 (poorly-differentiated) tumors being lower than the odds of making a correct diagnosis on grade 1 (well-differentiated) tumors (OR = 0.157, 95% CI 0.036–0.676). These findings are expected, as grade 1 tumors exhibit morphologic features characteristic of their tissue or cell type of origin, whereas grade 3 tumors typically do not.

### Impact of model accuracy on pathologist diagnostic accuracy

To confirm that the deep learning model was having the expected effect on pathologist accuracy, we explored how the accuracy of pathologists was affected when the model’s predictions were correct versus incorrect (see the “Statistical analyses” section of Methods for details). When the model’s prediction was correct (Table 3), pathologists reviewing a case with assistance had about 4.3 times the odds of making a correct final diagnosis, compared with the same pathologists reviewing the case without assistance (*p* = 0.000, OR = 4.289, 95% CI 2.360–7.794, fit to 1482 observations). However, when the model’s prediction was wrong, pathologists reviewing a case with assistance had less than one-third the odds of making a correct final diagnosis, compared with the same pathologists reviewing the case without assistance (*p* = 0.000, OR = 0.253, 95% CI 0.126–0.507, fit to 278 observations). These effects held across all pathologist experience levels and case difficulty levels.

### Performance of the model alone

For each WSI in the independent test set, a slide-level model prediction specific to each pathologist was generated by taking the average of the individual patch-level probabilities for the patches uploaded by the pathologist. These were converted to a binary prediction, using a probability threshold of 0.5. The diagnostic accuracy of the model was computed via comparison to the ground-truth labels, with 95% confidence intervals calculated using the *t*-score method. Using this method, the model alone achieved a mean accuracy of 0.842 (95% CI 0.808–0.876) on the independent test set.

## Discussion

In this study, we developed a deep learning algorithm intended to assist pathologists with the task of distinguishing between HCC and CC on H&E-stained WSI of primary liver tumors. Using a crossover experimental design with an independent test dataset, we evaluated the assistant’s impact on the diagnostic performance of 11 pathologists of different expertise levels, and observed (1) no statistically significant increase in mean accuracy with assistance, for a group of 11 pathologists, (2) a significant increase in accuracy with assistance, for the subset of nine pathologists of well-defined experience levels (GI specialists, non-GI specialists, and trainees), and (3) a significant differential impact of the model’s accuracy on the pathologists’ accuracy in the assisted state, when the model’s predictions were correct versus incorrect.

To date, few studies have prospectively evaluated the impact of AI assistance on pathologist diagnostic performance.^19^ To our knowledge, this is the only machine learning model to address the particular task of distinguishing between HCC and CC, and to explore the impact of model assistance across different experience and case difficulty levels. Seldom have these two factors been taken into account in studies of computer-assisted diagnosis. Most studies do not explicitly describe the distribution of easy versus difficult cases in the datasets used to validate model performance. When models are tested on datasets comprised of predominantly (or all) easy cases, and their performance compared with that of less experienced diagnosticians, this may lead to an overestimation of model performance and clinical utility, with subsequent poor generalizability to datasets containing the more challenging cases encountered in clinical practice, as well as less observed benefit in the hands of more experienced diagnosticians. In our study, all of the pathologists had lower accuracy on more challenging (grade 3) cases, in both the unassisted and assisted states, and the accuracy of the GI specialists was higher than that of the non-GI specialists and trainees, as would be expected. We found that, for the subset of nine pathologists with well-defined experience levels, assistance significantly improved diagnostic accuracy, regardless of particular experience level or case difficulty level. If these findings are reproduced in larger studies, they would suggest that the potential exists for AI assistance to significantly increase accuracy on specific, well-defined subspecialty diagnostic tasks.

An unexpected finding was the improvement in accuracy with assistance, despite the observation that the model alone did not perform as well as the pathologists alone (84 versus 88% overall accuracy on the independent test set, respectively). There are several possible explanations for this discrepancy. The accuracy of the model alone was calculated based on a relatively low binarization probability threshold of 0.5 on the model’s output, which may have resulted in a wider margin of error. This binarization threshold should not have impacted the pathologists’ diagnostic decision in the assisted mode, as the pathologists were provided with the actual probabilities output by the model (rather than a binarized diagnosis based on a 0.5 probability cutoff), along with class activation maps. Another possible explanation is that, since the pathologists were forced to input at least one image patch into the model, and to review the model’s output before making their diagnosis in the assisted mode, they might have taken less care in selecting patches for cases where they felt the diagnosis was obvious (e.g., when the model’s assistance was “unnecessary”). In such cases, this might have resulted in a selection bias for patches which were less discriminative for the diagnostic task at hand, resulting in a lower accuracy for the model alone. A third possible explanation is that the diagnosis made by the model was based on evaluation of only a few image patches (rather than the entire WSI), so the model may not have been exposed to more global, higher-level features that were appreciated by the pathologists, which helped them arrive at a correct diagnosis. Overall, the observation that the combination of the model and pathologist outperformed both the model alone, and the pathologist alone, suggests that the model and the pathologist are complementary, rather than parallel, with respect to the features each uses to arrive at a correct diagnosis, and that the model should be used to augment, rather than replace, the pathologist. Given the proliferation of recent studies reporting that an AI model is capable of outperforming, or even replacing, human diagnosticians, we feel that this is a finding which should not be ignored.

Another important observation from our experiment was that, while correct model predictions had a significant positive impact on pathologist accuracy in the assisted state, incorrect model predictions had an equally significant negative impact on accuracy. A review of the experimental data suggests that the pathologists might have been more strongly influenced by the model’s output on more difficult, compared with easier, cases. On grade 3 cases, the unassisted accuracy of the pathologists was 0.76 (95% CI 0.676–0.833). On the subset of grade 3 cases where the model’s prediction was correct, the pathologists’ assisted accuracy was 0.947 (95% CI 0.870–0.979). However, on the subset of grade 3 cases where the model’s prediction was incorrect, the pathologists’ assisted accuracy was only 0.310 (95% CI 0.185–0.480)—far lower than their unassisted accuracy. In contrast, for the diagnostically more straightforward cases (grades 1 and 2) the pathologists’ unassisted accuracy was 0.917 (95% CI 0.895–0.934), while their assisted accuracies were 0.982 (95% CI 0.969–0.990) and 0.654 (95% CI 0.558–0.738) when the model’s predictions were correct and incorrect, respectively. These results suggest that the pathologists might have relied more heavily on the model’s output for difficult cases. Overall, the finding that inaccurate model predictions can have a strong negative impact, even on subspecialty pathologists with particular expertise at the diagnostic task in question, raises concerns about the unintended effects of decision support tools, such as automation bias.^29^

As pathology specimen volumes continue to increase worldwide due to population growth, population aging, and the increasing prevalence and incidence of cancer and other diseases,^30,31^ there will be a greater need for novel tools to mitigate the adverse effects of a relative pathologist shortage. As diagnostic, prognostic, and predictive classifications of disease become ever more complicated, new tools will also be necessary to improve the accuracy and efficiency of current members of the pathologist workforce. The advent of digital pathology, the agreement on standards for image exchange, and improvements in slide scanning technology, WSI-viewing software, and image analysis algorithms have already begun to transform pathology, in much the same way that radiology has been transformed over the last two decades. However, most pathology practices still do not have access to digital slide scanners for the creation of WSI, or the resources to integrate non-vendor-proprietary image analysis algorithms into existing vendor-proprietary digital slide viewers. By designing our assistant as a web-based diagnostic tool that works on user-uploaded image patches, rather than WSI (each of which can be up to several gigabytes in size), we hoped to provide an efficient and accessible solution for the majority of pathologists, who might have access to only a microscope with a digital camera and an internet connection. Diagnostic assistants of the type tested in this study might provide particular benefit to pathology trainees during case preview, as well as pathologists working in low-resource settings with limited access to subspecialty expertise or ancillary diagnostic modalities. The adoption of such non-WSI-based assistance tools might lower the barrier to the incorporation of AI into clinical diagnostic workflows, where the turnaround time associated with using additional technology is important. However, consideration must be given to optimizing model performance, as well as thoughtful design of user interfaces, in order to minimize unintended negative consequences of computer assistance, such as automation bias.

This study has several limitations. The experimental setup was not entirely reflective of a real world practice setting, in which the pathologists would also have had access to corresponding clinical history, radiologic studies, laboratory findings (e.g., an elevated alpha fetoprotein level to suggest HCC), prior pathology reports, and additional immunohistochemistry to assist in the distinction between HCC and CC. The study also did not address cases of combined CC-HCC, due to the rarity of cases. Ideally, a diagnostic assistant would be able to identify not only cases of pure HCC or CC, but also cases where both subtypes are present within the same tumor. In addition, because the assistant required user input in the selection of image patches, the assistance was not fully automated. Given the inherent variability in user patch selection, future work might explore whether a model that runs on entire WSI, or one that incorporates automated patch selection, might produce a more consistent effect on pathologist performance across different experience levels. Finally, our study was limited to 70 TCGA WSI for model development and internal validation, and 80 Stanford WSI for external validation; this was largely due to the relative scarcity of CC cases, which comprise, on average, only 15% of primary liver cancers (compared with HCC, which comprises 70%), as well as the decision to maintain class balance between HCC and CC cases across the datasets. Further studies incorporating larger datasets from multiple hospitals and practice settings, as well as more pathologists with a broader range of backgrounds, will be necessary to validate our results.

In summary, we have developed and tested an AI tool to help pathologists with the subspecialty diagnostic task of distinguishing between the two most common primary liver cancers, HCC and CC. We found that, for a subset of 9 (out of 11) study pathologists, use of the tool significantly increased diagnostic accuracy, and that the accuracy of the assisted pathologists exceeded that of both the model alone, and the same pathologists without assistance. These findings suggest an optimistic future in which AI models might be used to augment, rather than replace, pathologists for certain subspecialty diagnostic tasks. At the same time, we also observed the potential for incorrect model predictions to mislead even expert subspecialty pathologists, resulting in significantly decreased diagnostic performance. Our results highlight the challenges of translating AI models into the clinical setting, and encourage those working in the field to consider the potential unintended effects of model assistance when designing and testing medical AI-assistance tools.

## Methods

### Datasets

Digital WSI of H&E-stained slides from formalin-fixed, paraffin-embedded (FFPE) primary hepatic tumor resections, from adult patients (aged 18 and older) with a pathologic diagnosis of either HCC or CC, formed the datasets for the study. Cases of combined CC-HCC and cases with poor slide preparation quality (presence of extensive artifact, such as tissue folds) were excluded. One representative tumor WSI per unique patient was included in the study. All data for the training, tuning, and validation datasets used for model development were obtained from the Cancer Genome Atlas’ (TCGA) hepatocellular carcinoma (LIHC) and cholangiocarcinoma (CHOL) diagnostic FFPE WSI collections.^24^ The independent test dataset was obtained from the slide archive of the Department of Pathology at Stanford University Medical Center.

For model development, a total of 70 WSI (35 HCC and 35 CC) were randomly selected from the TCGA-LIHC and TCGA-CHOL FFPE diagnostic slide collections, and randomly partitioned into training, tuning and validation datasets. The training dataset (20 WSI) was used to learn model parameters, the tuning dataset (24 WSI) was used to choose hyperparameters, and the validation dataset (26 WSI) was used to assess the model’s generalizability to previously unencountered data (Supplementary Fig. 1). Within each of these datasets, a 50:50 distribution of HCC and CC WSI was maintained.

The independent test dataset consisted of 80 representative WSI (40 CC and 40 HCC) of FFPE tumor tissue sections, one each from 80 unique patients randomly selected from a pool of all patients with HCC (250 patients) or CC (74 patients) who underwent surgical resection (partial or total hepatectomy) at Stanford University Medical Center in the years 2011–2017, and had glass slides available for retrieval from the pathology department archive. A 50:50 distribution of HCC and CC WSI was maintained for this dataset as well.

All WSI used in the study were in the SVS file format, scanned at ×40 magnification (0.25 µm per pixel) on an Aperio AT2 scanner (Leica Biosystems, Nussloch, Germany). The reference standard diagnosis for all examinations was confirmed by a U.S. board-certified, GI/liver fellowship-trained pathologist at Stanford University Medical Center (J.S.). All cases in the independent test dataset had been reviewed by a GI/liver subspecialty pathologist at the time of the original diagnosis, with evaluation of additional immunohistochemical and special stains, as well as intradepartmental consensus review, performed for diagnostically challenging cases. Confirmation of the original diagnoses entailed re-review of all available H&E and immunostained slides from each case by the reference pathologist, as well as review of the original pathology report and all supporting clinical, radiologic, molecular diagnostic, and other ancillary findings. For the TCGA dataset, confirmation of the diagnoses entailed re-review of all available H&E whole-slide images from each case. All relevant accompanying metadata available through the TCGA’s Genomic Data Commons (GDC), including the original pathology reports (PDF files) containing the results of any additional immunohistochemical or special stains performed, as well as available radiologic, molecular diagnostic, and clinical history, were re-reviewed for each case. No diagnostic discrepancies were identified between the original diagnosis and the reference pathologist’s diagnosis, for either the TCGA or Stanford datasets.

### Model development

We trained a convolutional neural network (CNN), a type of neural network which is particularly effective on image data, to classify image patches as containing either HCC or CC. Inspired by the organization of neurons in the human visual cortex, a CNN takes advantage of a parameter-sharing receptive field to learn local features of an image.^25^ The specific architecture used was a densely-connected CNN (DenseNet), in which each layer in the network is connected to every other layer in a feed-forward fashion.^26^ Due to the large sizes of the WSI files, which precluded inputting entire WSIs into the model, model training and evaluation were performed using image patches extracted from the WSI. For each WSI in the training, tuning, and validation datasets, all tumor-containing regions were annotated by the reference pathologist. From these tumor regions, 1000 square image patches of size 512 × 512 pixels (128 × 128 µm) were randomly sampled (see Supplementary Fig. 2), yielding a total of 20,000 image patches for model training. For the tuning and internal validation datasets, we randomly extracted 100 similarly sized image patches per WSI, for a total of 2400 tuning patches and 2600 validation patches. Pixel values were normalized using the mean and standard deviation of the pixel values from the TCGA training set. These normalized patches then served as the input to the CNN. For additional details regarding image pre-processing and model development, please see the Supplementary materials.

### Pathologist experiment

Eleven pathologists at Stanford University Medical Center were recruited for the study, classified into the following four experience level subgroups: (i) three GI pathologists who had spent the last 3 or more years diagnosing HCC and CC in daily independent practice (8, 8, or 35 years of individual practice experience, respectively), (ii) three non-GI subspecialty pathologists with at least 12 years of independent practice experience (16, 25, or 29 years of individual practice experience, respectively) and no diagnostic exposure to HCC or CC for the last 5 or more years, (iii) three Anatomic Pathology residents (trainees) without independent diagnostic experience, but with some limited exposure to HCC and CC cases as part of their training within the last 2 years (one second-year resident and two third-year residents), and (iv) two pathologists who otherwise did not fall within the criteria for the previous three subgroups, and were assigned to the pathologist, not otherwise-classified (NOC) subgroup. Specifically, the two pathologists in the last category were non-GI pathologists with 1 year of independent practice experience (one in Dermatopathology, and the other in Cytology/Head and Neck Pathology), and recent general surgical pathology fellowship training, during which time they had exposure to HCC/CC cases. One of the two pathologists was also practicing part-time in a general pathology practice.

The ObjectiveView digital pathology image viewer (2018 Objective Pathology Services, Ontario, Canada) was used by the pathologists to navigate the WSI during the experiment. Prior to the start of each diagnostic session, the participants were given time to familiarize themselves with ObjectiveView and the assistant, using a brief tutorial document and four practice WSI (separate from the 80 test WSI). They practiced digital slide navigation, image patch selection, and use of the assistant. For each practice WSI, the pathologists first were informed of the ground-truth (HCC or CC), then selected different tumor ROIs at ×10 objective magnification, of ~2000 × 2000 pixels, for upload to the diagnostic assistant, and finally viewed the model’s generated probability and CAM for each selected patch. The same desktop workstation setup, consisting of (1) a 2017 iMac Pro with 27″ Retina 5K display, with ObjectiveView and the assistant open concurrently for WSI navigation/image patch selection and uploading of image patches, respectively, and (2) an adjacent desktop PC running Windows 10, for entering diagnoses into the web survey, was used to perform all experiments. Each session was monitored by an administrator, who was present for the duration of each experiment. The study was approved by the Stanford University Institutional Review Board, with waived informed consent obtained for inclusion of the 80 WSI used in the experiment.

### Statistical analyses

The diagnostic accuracy of each group of pathologists, with and without assistance, was assessed on the independent test set. The average accuracy across all four subgroups was also computed. Confidence intervals were calculated using the Wilson score method.^32^

Sensitivity and specificity for each pathologist was generated using the caret package in R.^33^

Mixed-effect multivariate logistic regression models were developed to evaluate whether assistance produced a significant difference in accuracy, as well as to explore how tumor grade (a proxy indicator of case difficulty level) and experience level affected diagnostic accuracy. Pathologists and slides were included in the model as random effects, with the assistance status (assisted versus unassisted), tumor grade, and pathologist experience subgroup included as fixed effects. To evaluate the impact of a particular fixed effect of interest on diagnostic accuracy, the full model was compared with the same model with the fixed effect of interest left out, using a likelihood ratio Chi-square test. To further explore the impact of model accuracy on the pathologists’ diagnostic accuracy, we separately fit the same mixed-effect model to (1) the subset of cases on which the model’s predictions were correct, and (2) the subset of cases on which the model’s predictions were incorrect. For each subset, the likelihood ratio Chi-square test was again used to compare the full model to the same model without the assistance status. All mixed-effect models were developed using the lme4 package in R.^34^ The Benjamini–Hochberg (BH) correction was applied to account for multiple hypothesis testing when appropriate, with a BH-adjusted *p*-value ≤ 0.05 (two-tailed) indicating statistical significance.

### Reporting summary

Further information on research design is available in the Nature Research Reporting Summary linked to this article.

## Supplementary information

Supplementary Information

Reporting Summary

## Data Availability

All whole-slide images used for model development and internal validation are publicly available online through the TCGA’s Genomic Data Commons (https://portal.gdc.cancer.gov/). The exact case IDs used for model development are openly accessible at 10.5281/zenodo.3625234. The Stanford whole-slide image dataset is not publicly available, in accordance with institutional requirements governing human subject privacy protections. However, the de-identified image patches selected by the participants during the pathologist experiment are available, also at 10.5281/zenodo.3625234. The results from the pathologist experiment used for statistical analyses and generation of manuscript figures/tables are available at https://github.com/stanfordmlgroup/lca-code/tree/master/paper.

## Code availability

Code for the neural network, CAM generation, and statistical analyses reported in this paper is freely available at https://github.com/stanfordmlgroup/lca-code.
