# Supplementary material for: Impact of a deep learning assistant on the histopathologic classification of liver cancer
Source: NPJ Digit Med. 2020 Feb 26;3:23. doi: 10.1038/s41746-020-0232-8 (PMC7044422; doi:10.1038/s41746-020-0232-8)
Supplement: Supplementary file 1 — Supplementary Information [file 41746_2020_232_MOESM1_ESM.pdf]

Supplementary Notes

Model Architecture

The model was built on a pretrained 121-layer DenseNet architecture. The DenseNet architecture is a convolutional neural network consisting of blocks of convolutional layers, such that each layer is directly connected to every other layer within a block. This facilitates the gradient flow through the network during training, making it easier to train deeper, more complicated networks. We replaced the final, fully-connected softmax layer with a sigmoid layer with a single output for our binary classification task.

Model Training

The deep learning process consisted of feeding training images to the network, receiving a prediction from the network, and iteratively updating the parameters to decrease the prediction error, which was computed by comparing the network's prediction to the ground truth label for each image. By performing this procedure using a representative set of images, the resulting network could make predictions on previously unencountered H&E-stained histopathology images. The weights of the network were initialized to those from a model pretrained on ImageNet, a large image classification dataset.[1] The model was trained end-to-end, using stochastic gradient descent with a momentum of 0.9, on mini-batches of size 10. We used a step-based scheduler, which decayed the learning rate by a factor of 0.1 every 20,000 iterations. Learning rates were randomly sampled between 1e-4 and 1e-7. To improve the generalizability of the models, several forms of data augmentation were used during training, including rotations and flips of the input images.

Model Selection

Model selection consisted of three steps. First, 50 networks with randomly sampled hyperparameters were trained on the TCGA training dataset, and evaluated on the tuning set. From these, the 10 best-performing networks were selected and evaluated on the internal validation set, to assess generalizability to unencountered data. The network with the highest accuracy on the internal validation set was used to create the assistant. The model selection process is summarized in Supplementary Figure 1.

Assistant Web Application Architecture

The assistant's web architecture is comprised of an HTML5 front end and a Python back end. The front end communicates with the back end via a JSON-based REST interface. The front end is responsible for authenticating the users and allowing them to upload patches, view the model's output probabilities and explanatory CAMs in real time, and provide feedback regarding the model's output.

Model Explanations

Class activation maps (CAMs) were used to highlight regions with the greatest influence on the model's decision (see Supplementary Figure 4). For a given patch, the CAM was computed for both classes (HCC and CC) by taking the weighted average across the final convolutional feature map, with weights determined by the linear layer. The CAM was then scaled according to the output probability, so that more confident predictions appeared brighter. Finally, the map was upsampled to the input image resolution, and overlaid onto the input image.

Supplementary References

1. J. Deng, W. Dong, R. Socher, L. Li, Kai Li and Li Fei-Fei, "ImageNet: A large-scale hierarchical image database," *2009 IEEE Conference on Computer Vision and Pattern Recognition*, Miami, FL, 2009, pp. 248-255. doi: 10.1109/CVPR.2009.5206848

Supplementary Figures

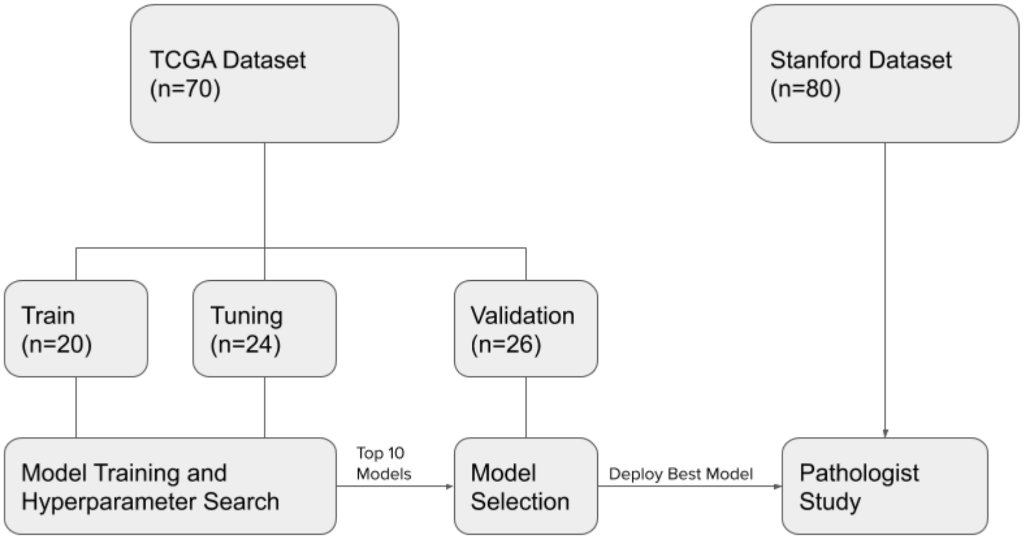

Supplementary Figure 1. Model development and selection
Fifty models were trained with randomly selected hyperparameters. The ten best-performing models on the tuning set were evaluated on the validation set to assess their generalizability. The model with the highest accuracy on the validation set was deployed in the assistant, and evaluated during the pathologist experiment on the independent test (Stanford) dataset.

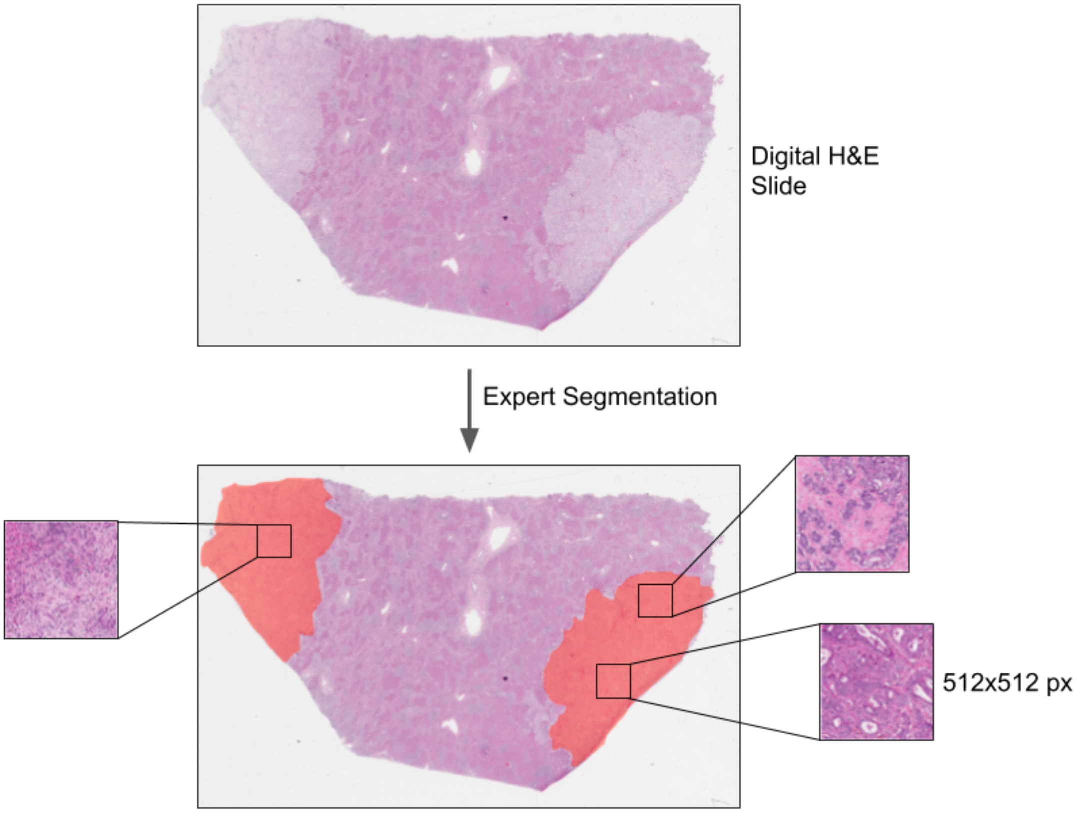

Supplementary Figure 2. Data preprocessing
The model was trained on 512 x 512 pixel patches, which were randomly sampled from tumor regions segmented by the reference GI pathologist. The sample WSI depicts segmented tumor regions (red), with three randomly sampled patches (patches not drawn to scale). A total of 1,000 training patches were sampled from each WSI.

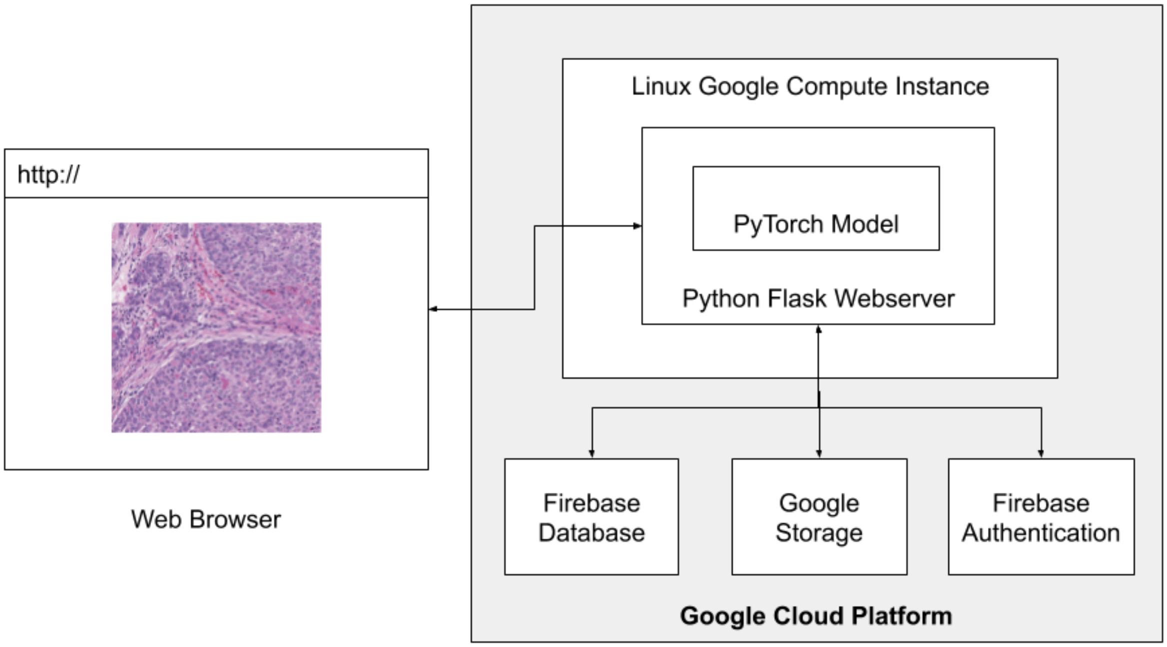

Supplementary Figure 3: Web Architecture
The assistant's web architecture is comprised of an HTML5 front end and a Python back end. The front end communicates with the back end via a JSON-based REST interface. The front end is responsible for authenticating the users and allowing them to upload patches, view the model's results and explanatory CAMs in real time, and provide feedback about the model's output.

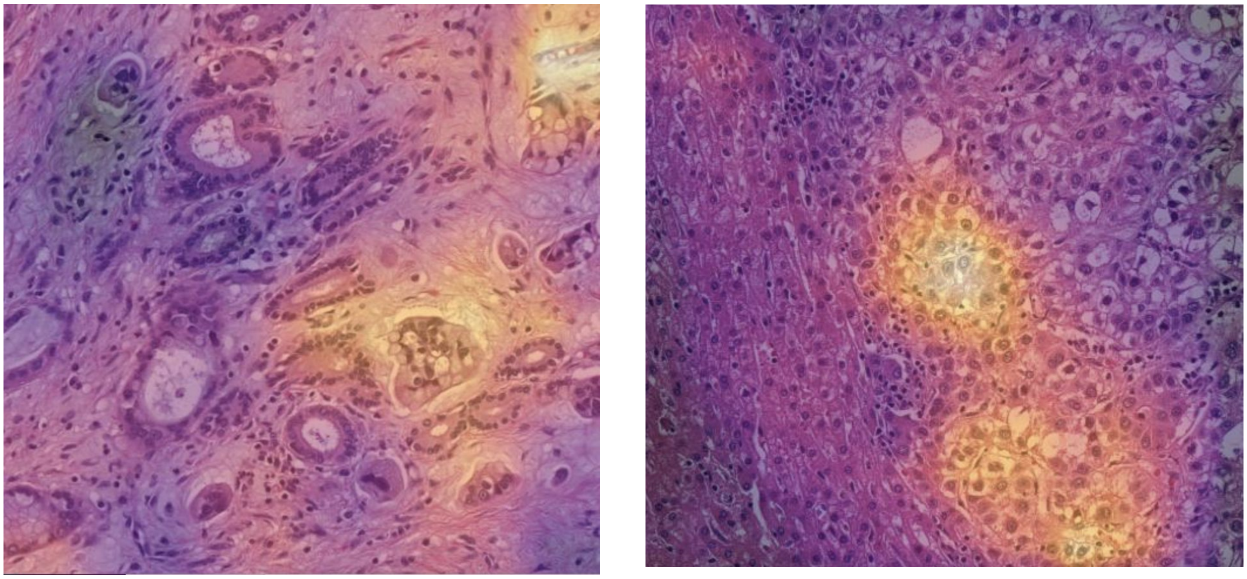

(a) (b)

Supplementary Figure 4. Example class activation maps (CAMs) for cholangiocarcinoma and hepatocellular carcinoma. a. the patch on the left was correctly classified as cholangiocarcinoma with 95.6% confidence. **b.** the patch on the right was correctly classified as hepatocellular carcinoma with 99.7% confidence.

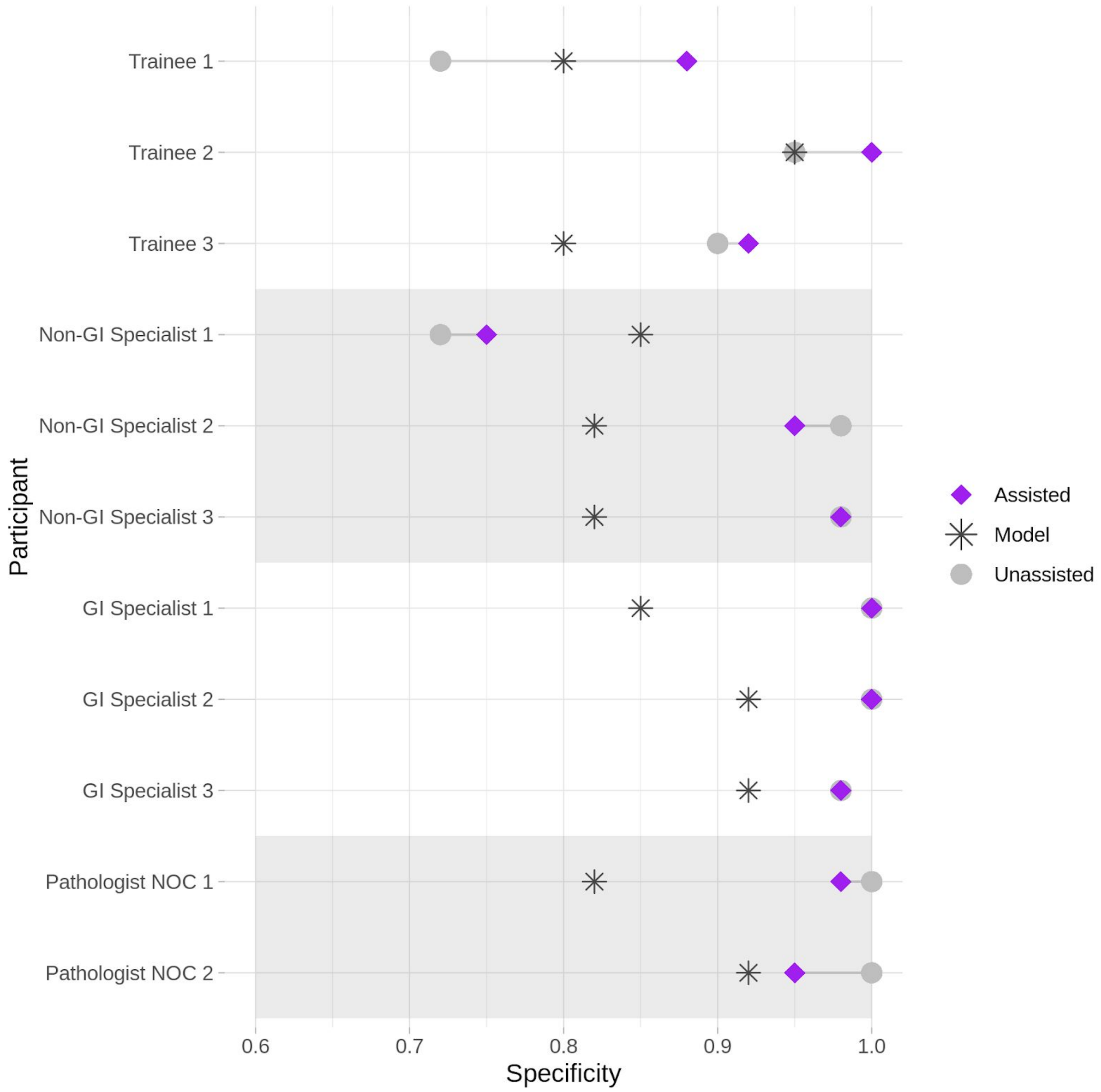

Supplementary Figure 5. Diagnostic specificities for individual pathologists, with and without assistance, as well as for the model alone (based on patches input by the pathologists)

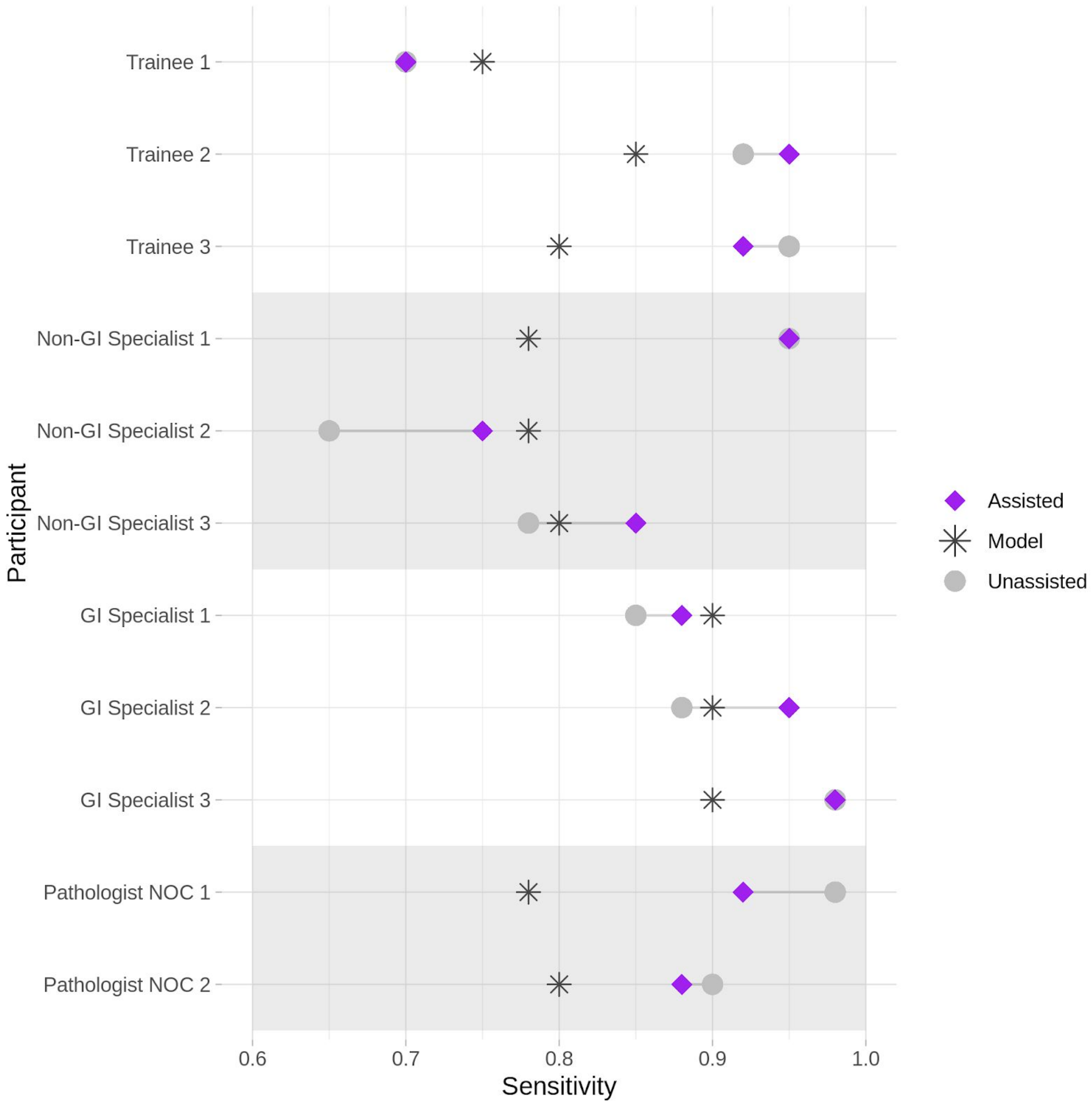

Supplementary Figure 6: Diagnostic sensitivities for individual pathologists, with and without assistance, as well as for the model alone (based on patches input by the pathologists)

Supplementary Tables

Supplementary Table 1. Average diagnostic accuracies, sensitivities, and specificities for individual pathologists, with (Asst) and without (Unasst) assistance, as well as for the model alone (Algo)

Pathologist	Accuracy (95% CI)			Specificity (95% CI)			Sensitivity (95% CI)		
	Algo	Unasst	Asst	Algo	Unasst	Asst	Algo	Unasst	Asst
Trainee 1	0.78 (0.67, 0.85)	0.71 (0.61, 0.80)	0.79 (0.69, 0.86)	0.80 (0.65, 0.90)	0.72 (0.57, 0.84)	0.88 (0.74, 0.95)	0.75 (0.60, 0.86)	0.70 (0.55, 0.82)	0.70 (0.55, 0.82)
Trainee 2	0.90 (0.81, 0.95)	0.94 (0.86, 0.97)	0.97 (0.91, 0.99)	0.95 (0.83, 0.99)	0.95 (0.83, 0.99)	1.00 (0.91, 1.00)	0.85 (0.71, 0.93)	0.93 (0.80, 0.97)	0.95 (0.83, 0.99)
Trainee 3	0.80 (0.70, 0.87)	0.93 (0.85, 0.97)	0.93 (0.85, 0.97)	0.80 (0.65, 0.90)	0.90 (0.77, 0.96)	0.93 (0.80, 0.97)	0.80 (0.65, 0.90)	0.95 (0.83, 0.99)	0.93 (0.80, 0.97)
Non-GI Specialist 1	0.81 (0.71, 0.88)	0.84 (0.74, 0.90)	0.85 (0.76, 0.91)	0.85 (0.71, 0.93)	0.72 (0.57, 0.84)	0.75 (0.60, 0.86)	0.78 (0.62, 0.88)	0.95 (0.83, 0.99)	0.95 (0.83, 0.99)
Non-GI Specialist 2	0.80 (0.70, 0.87)	0.81 (0.71, 0.88)	0.85 (0.76, 0.91)	0.82 (0.68, 0.91)	0.97 (0.87, 1.00)	0.95 (0.83, 0.99)	0.78 (0.62, 0.88)	0.65 (0.50, 0.78)	0.75 (0.60, 0.86)
Non-GI Specialist 3	0.81 (0.71, 0.88)	0.88 (0.78, 0.93)	0.91 (0.83, 0.96)	0.82 (0.68, 0.91)	0.97 (0.87, 1.00)	0.97 (0.87, 1.00)	0.80 (0.65, 0.90)	0.78 (0.62, 0.88)	0.85 (0.71, 0.93)
GI Specialist 1	0.88 (0.78, 0.93)	0.93 (0.85, 0.97)	0.94 (0.86, 0.97)	0.85 (0.71, 0.93)	1.00 (0.91, 1.00)	1.00 (0.91, 1.00)	0.90 (0.77, 0.96)	0.85 (0.71, 0.93)	0.88 (0.74, 0.95)
GI Specialist 2	0.91 (0.83, 0.96)	0.94 (0.86, 0.97)	0.97 (0.91, 0.99)	0.93 (0.80, 0.97)	1.00 (0.91, 1.00)	1.00 (0.91, 1.00)	0.90 (0.77, 0.96)	0.88 (0.74, 0.95)	0.95 (0.83, 0.99)
GI Specialist 3	0.91 (0.83, 0.96)	0.97 (0.91, 0.99)	0.97 (0.91, 0.99)	0.93 (0.80, 0.97)	0.97 (0.87, 1.00)	0.97 (0.87, 1.00)	0.90 (0.77, 0.96)	0.97 (0.87, 1.00)	0.97 (0.87, 1.00)
Pathologist NOC 1	0.80 (0.70, 0.87)	0.99 (0.93, 1.00)	0.95 (0.88, 0.98)	0.82 (0.68, 0.91)	1.00 (0.91, 1.00)	0.97 (0.87, 1.00)	0.78 (0.62, 0.88)	0.97 (0.87, 1.00)	0.93 (0.80, 0.97)
Pathologist NOC 2	0.86 (0.77, 0.92)	0.95 (0.88, 0.98)	0.91 (0.83, 0.96)	0.93 (0.80, 0.97)	1.00 (0.91, 1.00)	0.95 (0.83, 0.99)	0.80 (0.65, 0.90)	0.90 (0.77, 0.96)	0.88 (0.74, 0.95)

Supplementary Table 2. Results of the pathologist experiment, with univariate association of diagnostic accuracy with individual predictors, with or without assistance (1,760 observations)

Variable (predictor)	Diagnostic accuracy							
	With assistance				Without assistance			
	Correct n (%)	Incorrect n (%)	Total n (%)	*p*	Correct n (%)	Incorrect n (%)	Total n (%)	*p*
Experience level:								
GI pathologist	231 (96)	9 (4)	240 (100)	0.002***	227 (95)	13 (5)	240 (100)	0.000***
Non-GI pathologist	209 (87)	31 (13)	240 (100)		202 (84)	38 (16)	240 (100)	
Trainee	215 (90)	25 (10)	240 (100)		206 (86)	34 (14)	240 (100)	
Pathologist NOC	149 (93)	11 (7)	160 (100)		155 (97)	5 (3)	160 (100)	
Ground truth								
HCC	415 (94)	25 (6)	440 (100)	0.002**	409 (93)	31 (7)	440 (100)	0.002 **
CC	389 (88)	51 (12)	440 (100)		381 (87)	59 (13)	440 (100)	
Tumor grade								
1: well-diff.	104 (95)	6 (5)	110 (100)	0.000***	102 (93)	8 (7)	110 (100)	0.000***
2: moderately-diff.	618 (94)	42 (6)	660 (100)		604 (92)	56 (8)	660 (100)	
3: poorly-diff.	82 (75)	28 (25)	110 (100)		84 (76)	26 (24)	110 (100)	
Pathologist diagnosis:								
1=HCC	415 (89)	51 (11)	466 (100)	0.010 *	409 (87)	59 (13)	468 (100)	0.013 *
0=CC	389 (94)	25 (6)	414 (100)		381 (92)	31 (8)	412 (100)	
Model error								
Yes	79 (57)	60 (43)	139 (100)	0.000***	105 (76)	34 (24)	139 (100)	0.000***
No	725 (98)	16 (2)	741 (100)		685 (92)	56 (8)	741 (100)	
Total	804 (91)	76 (9)	880 (100)		790 (90)	90 (10)	880 (100)	

* $p \leq 0.05$; ** $p \leq 0.01$; *** $p \leq 0.001$

Note: Total percentages may not add up to 100%, due to rounding error. The unit n corresponds to a single observation (e.g. one whole-slide image read). Pathologist diagnosis = final diagnosis entered on a given WSI by the pathologist during the experiment. Model error = whether the model's prediction was wrong (based on the patch(es) input by each pathologist

during the assisted mode), compared with the ground truth. The p-values listed above are from 10 individual Pearson Chi-square tests of association (or Fisher's exact tests, when appropriate) between accuracy and the individual predictor. No post-hoc subgroup (pairwise) analyses were performed (for example, between GI pathologists and Pathology trainees under Experience level). All significance levels are two-tailed.

Supplementary Table 3. Results of mixed-effect logistic regression analyses evaluating the impact of individual predictors (fixed effects) on diagnostic accuracy prediction, for all pathologists (11 pathologists, 1,760 observations)

**Predictor**	**Diagnostic Accuracy (Correct vs. Incorrect)**		
	**Odds ratio**	**95% Confidence interval**	***p***
Computer assistance: Y vs. N	1.281	(0.882, 1.862)	0.184
Experience level:			
Non-GI vs. GI specialist	0.204	(0.082, 0.508)	0.005 **
Trainee vs. GI specialist	0.299	(0.119, 0.753)	
Path. NOC vs. GI specialist	0.949	(0.318, 2.834)	
Tumor grade:			
Grade 2 vs. Grade 1	0.783	(0.239, 2.571)	0.010*
Grade 3 vs. Grade 1	0.157	(0.036, 0.676)	

* $p \leq 0.05$; ** $p \leq 0.01$; *** $p \leq 0.001$

Note: The p-values in this table represent the results of individual likelihood ratio Chi-square tests (one for each of the three fixed effects). All significance levels are two-tailed.

Supplementary Table 4. Results of mixed-effect logistic regression analyses evaluating the impact of individual predictors (fixed effects) on diagnostic accuracy prediction, for pathologists of well-defined experience levels (9 pathologists, 1,440 observations)

**Predictor**	**Diagnostic Accuracy (Correct vs. Incorrect)**		
	**Odds ratio**	**95% Confidence interval**	***p***
Computer assistance: Y vs. N	1.499	(1.007, 2.230)	0.045*
Experience level:			
Non-GI vs. GI specialist	0.203	(0.079, 0.523)	0.009 **
Trainee vs. GI specialist	0.298	(0.114, 0.778)	
Tumor grade:			
Grade 2 vs. Grade 1	0.776	(0.235, 2.565)	0.013*
Grade 3 vs. Grade 1	0.159	(0.036, 0.691)	

* $p \leq 0.05$; ** $p \leq 0.01$; *** $p \leq 0.001$

Note: The p-values in this table represent the results of individual likelihood ratio Chi-square tests (one for each of the three fixed effects). All significance levels are two-tailed.
